# Construction of hematoxylin–eosin, immunohistochemistry, and EBER-ISH methodology after trichloroisocyanuric acid treatment in melanin-containing tissues

**DOI:** 10.1038/s41598-022-20535-7

**Published:** 2022-09-26

**Authors:** Chaoshan Wang, Xia Yang, Ting Wang, Ya Wang, Jiong Shi, Qi Sun, Yihua Wang, Hongyan Wu

**Affiliations:** Department of Pathology, Nanjing Drum Tower Hospital, The Affiliated Hospital of Nanjing University Medical School, 321 Zhongshan Road, Nanjing, China

**Keywords:** Biochemistry, Biological techniques, Biotechnology, Immunology, Medical research

## Abstract

This study investigated the effects of trichloroisocyanuric acid (TCCA) on the bleaching and morphology of melanin-containing pathological sections. The pathological sections of 27 patients with high melanin content were bleached with 0.5% potassium permanganate, 10% hydrogen peroxide, and different concentrations of TCCA. Significant differences were found among the blank control group, 1% TCCA group (*P* < 0.0001). The hematoxylin–eosin (HE) score of the "recovery pH" HE staining group after treatment with 1% TCCA was similar to that of the "Conventional HE" scheme group (*P* > 0.05). The morphological diagnostic scores of 50 cases of pathological sections with different melanin content before and after TCCA bleaching were compared. The results showed a significant difference in the diagnostic score between the middle- and high-melanin content groups before and after 1% TCCA bleaching (*P* < 0.05). Immunohistochemical staining was performed on meningeal melanoma tissue. For this, 8% TCCA solution was used to remove melanin after Ki67, S100, and β-catenin immunohistochemical staining. After bleaching with TCCA, the staining and positioning of each marker with different localization were accurate and the background was clear. The same results were also shown with EBER-ISH. This study concluded that 1% TCCA could be used for HE staining of pathological sections containing melanin, and "restore pH" HE scheme as the staining method after TCCA melanin removal. Further, 8% TCCA was used for bleaching after immunohistochemical DAB staining. Melanin can be completely removed, and sections can meet diagnostic needs.

## Introduction

Melanocytic nevus belongs to the category of benign tumors, while malignant melanoma is a highly aggressive malignancy. Malignant melanoma originates in the skin or in the central nervous system. The pathological diagnosis is often interfered with by melanin in tissues, which affects its diagnostic accuracy. Therefore, removing melanin from pathological sections, the morphology of tumor cells (such as cell karyotype, karyoplasmic ratio, and mitotic image), organization structure (such as mature phenomenon), and involvement range become clear. It may be of great help to pathological diagnosis and subsequent clinical decision-making, especially in cases involving differential diagnoses of benign and malignant tumors. This study used chlorine-containing disinfection tablets (trichloroisocyanuric acid, TCCA) to bleach the melanin in tissues and to clarify the application effect and value of TCCA in the pathological diagnosis of melanogenic disease and tumor.

Previous studies have shown that melanin can be identified and distinguished using different staining agents, such as Giemsa, methyl green, and ferrous sulfate^1–3^. The bleaching technology has been widely used since 1958. It is considered to be an effective solution to remove melanin using the principle of oxidant oxidation to break the phenol ring. Many kinds of oxidants are used, such as potassium permanganate, hydrogen peroxide, and chlorine. Among these, potassium permanganate has a strong depigmentation effect. However, the potassium permanganate–oxalic acid method is time-consuming (0.3–2 h) and involves multiple steps. Also, the oxidation antigen is damaged, affecting further diagnosis. Hydrogen peroxide has been widely reported in recent years^1,4,5^. For example, 10% hydrogen peroxide has the function of oxidation and bleaching. However, the bleaching time is as long as 12–48 h, even 2–3 weeks; it is almost difficult to bleach within 12 h, with no advantage in terms of time efficiency. We inadvertently found that chlorine-containing disinfection tablets or chlorine-containing effervescent tablets (trade name Deck Corning disinfection tablets) could remove traces of hematoxylin from the laboratory table because they contained TCCA, which was a strong oxidant. TCCA can be used as a bleaching agent in the printing and dyeing industry. It has the advantages of no damage to fibers and better bleaching efficiency compared with sodium hypochlorite and bleach. This phenomenon inspired us to conduct this study.

## Materials and methods

### Materials

This study selected 50 cases from the Department of Pathology, Nanjing Drum Tower Hospital, between 2018 and 2020, including 29 cases of melanoma, 17 cases of blue nevus and complex nevus, and 4 cases of melanocytoma. According to the proportion of melanin content in tumor tissue, 50 pathological sections with melanin were divided into three groups: low, medium, and high. Low–melanin content group (proportion of melanin area < 25%) comprised 12 cases, medium–melanin content group (proportion of melanin area: 25%–50%) comprised 11 cases, and high–melanin content group (proportion of melanin area > 50%) comprised 27 cases. Nine normal tissues were used as controls, including kidney, prostate, and pancreas et al. All the samples were fixed with 10% neutral buffered formalin for 24 h. The section thickness was 3 μm mounted on adhesion microscope sections.

### Reagent preparation

(1) 0.5% Potassium permanganate solution: A: potassium permanganate 31.6 mM; B: oxalic acid: 22.2 mM, used after mixing. (2) 10% Hydrogen peroxide solution 2.94 M. (3) TCCA: Disinfectant tablets 1, 2, 4, 8 and 16 g were dissolved in 100 ml of distilled water to obtain 1%, 2%, 4%, 8% and 16% concentrations of trichloroisocyanuric acid, respectively . (4) EDTA-Tris solution (pH 8–11): Tris 97.6 mM, EDTA-Tris 19.3 mM, pH 9.0. The pH was adjusted and labeled by adding HCl or NaOH. (5) 1%–30% Glacial acetic acid solution: The concentrations of glacial acetic acid were 1.67 mol/L, 3.34 mol/L, and 5.01 mol/L. (5) Immunohistochemistry related reagents: primary antigen such as Ki67, S100, β-catenin et al. The supporting testing system (No. k5007) was from Dako Agilent. (6) EBER-ISH: EBER UltraPATH test kit (No. ISH-7001UM) was from Origene.

### Experimental method

(1) TCCA melanin removal method: the sections were incubated in different concentrations in TCCA solution at room temperature (0.25%, 0.5%, 1%, 2%, 4%, and 8%). The pathological sections containing melanin were incubated with TCCA solution. At the beginning, the pathological sections were observed every 5 min. When the melanin was obviously lighter, the pathological sections were observed every 2 min. The bleaching time was recorded, and the sections were washed thoroughly. (2) 0.5% Potassium permanganate–2% oxalic acid melanin removal method: the sections were incubated with 0.5% potassium permanganate for 15 min, washed under running water, incubated with 2% oxalic acid for 1–2 min, and observed under the microscope. (3) Hydrogen peroxide melanin removal method: The sections were incubated with 10% hydrogen peroxide solution for 15 min to 72 h, observed under the microscope, and washed under running water (4) The quality problem of HE morphology is often related to pretreatment, and the oxidant is also an important factor^6^. Bleaching with strong oxidants may lead to the changes in intracellular and extracellular isoelectric points^7^. Conventional HE scheme was an automated process in routine work. "Restored pH" HE scheme was established to neutralize the changes in protein composition polarity caused by oxidants and to restore cell protein isoelectric points and staining polarity. The process of three HE staining methods is presented in Table 1. (5) Immunohistochemical staining and EBER-ISH: the pathological sections were dewaxed and hydrated, and then blocked with 3% H_2_O_2_ for 30 min and rinsed with distilled water. After that, the sections were immersed in EDTA-Tris buffer for high temperature and high pressure repair. The sections were placed loosely on the staining dish and the sections were allowed to cool for 20 min. The sections were rinsed with phosphate-buffered saline (PBS) for 2 × 2 min. The sections were then incubated with primary antibody (Ki67/S100/β-catenin et al.) for 1 h at room temperature. They were rinsed with PBS for 2 × 2 min and incubated with the secondary antibody for 20 min at room temperature. They were incubated with DAB at room temperature for 5 min, rinsed with PBS, and mounted with a coverslip. (6) EBER-ISH: After the pathological sections were processed, the experiments were performed by EBER UltraPATH test kit. (7) Melanin bleach with TCCA: After the sections were stained with immunohistochemical DAB, they were incubated and bleached with 8% TCCA solution. Under the microscope, the cells were transparent when no melanin residue remained. After hematoxylin staining for 1–2 min, the sections were dehydrated and mounted with a coverslip.

**Table 1:** Three HE staining methods.

Conventional HE scheme		Extended time HE scheme			Restored pH HE scheme		
Xylene I	2 min	Xylene I		2 min	Xylene I		2 min
Xylene II	2 min	Xylene II		2 min	Xylene II		2 min
Xylene III	2 min	Xylene III		2 min	Xylene III		2 min
100% ethanol	1.5 min	100% ethanol		1.5 min	100% ethanol		1.5 min
95% ethanol	1.5 min	95% ethanol		1.5 min	95% ethanol		1.5 min
85% ethanol	1.5 min	85% ethanol		1.5 min	85% ethanol		1.5 min
75% ethanol	1.5 min	75% ethanol		1.5 min	75% ethanol		1.5 min
Distilled water to wash	1 min	Distilled water to wash		1 min	Distilled water to wash		1 min
–		–			EDTA-Tris pH8.0		5 min
–		–			EDTA-Tris pH9.0		10 min
–		–			EDTA-Tris pH10.0		20 min
Hematoxylin staining	3.5 min	Hematoxylin staining		10 ~ 30 min	Hematoxylin staining		10 min
Bluing buffer	2.5 min	Bluing buffer		2.5 min	Back blue with warm water until the lymphocyte nucleus turns dark blue		
–		–			1% Glacial acetic acid		1 ~ 2.5 min
–		–			10% Glacial acetic acid		1 ~ 2.5 min
–		–			30% Glacial acetic acid		1 ~ 2.5 min
70% ethanol	30 s	70% ethanol		30 s	–		
Eosin staining	1 min	Eosin staining		10 ~ 30 min	Eosin staining		5 min
75% ethanol	1.5 min	75% ethanol		1.5 min	Dehydration with anhydrous ethanol		
85% ethanol	1.5 min	85% ethanol		1.5 min	Xylene transparency		
95% ethanol	1.5 min	95% ethanol		1.5 min	–		
Xylene I	2 min	Xylene I		2 min	–		
Xylene II	2 min	Xylene II		2 min	–		
Xylene III	2 min	Xylene III		2 min	–		
Mount with coverslip		Mount with coverslip			Mount with coverslip		

### Result interpretation

(1) The effective melanin region was selected for gray value analysis. The gray value data of each group was recorded, and the average score was calculated using ImagePro Plus 6. (2) Under the double-blind condition of two or more pathologists, the results of HE staining were evaluated with 0–100 points. If the corresponding standard was not met, the score was deducted. A score of less than 60 indicated incomplete diagnosis, and a score of 100 indicated complete diagnosis^1^. The scoring criteria are presented in Table 2.

**Table 2:** HE staining scoring rules.

HE staining scoring rules		
Nuclear clarity	40	Lymphocyte light blue (−20) Nucleolus chromatin indistinguishable (−20)
Cytoplasmic uniformity	30	Cytoplasmic staining light (−20) Red blood cell is not cherry red (−10)
Red blue contrast	20	Too light or too dark, poor contrast (−20)
Morphological integrity	10	Tissue folding and flaking (−5 to 10); all flaking (−10)
Total	100	Unqualified (< 60)

### Statistical analysis

SPSS 22.0 software and GraphPad Prism 8 were used for statistical analysis. The measurement data subject to normal distribution are expressed by means ± standard deviation, and the analysis of variance is performed for comparison between groups; Statistical significance was calculated by Fisher's exact test. False discovery rate (FDR) corrected P-values less than 0.05 were considered statistically significant.

### Ethics approval and consent to participate

The study adhered to the Declaration of Helsinki and was approved by the National Regional Committee for Medical and Health Research Ethics, and registered with the Ethics Committee of Drum Tower Hospital Affiliated to Medical College of Nanjing University (2017L00611). Written informed consent was obtained from all participants prior to any study-related procedure.

## Results

### Bleaching concentration of TCCA

Three pathological sections with a high melanin content were incubated with TCCA at room temperature (22–25 °C). The mean bleaching time corresponding to the concentrations of 0.25%, 0.5%, 1%, 2%, 4%, and 8% was 138.30 ± 7.64, 61.67 ± 5.77, 14.33 ± 0.58, 12.0 ± 1.00, 7.33 ± 0.58, and 5.67 ± 0.58 min, respectively. When the effective concentration reached 1%, the bleaching time could be shortened significantly. When the experiment was repeated, an occasional desquamate phenomenon was observed at a concentration above 2%. Considering time efficiency, operation simplicity, and cost performance, the reproducible condition was established to be 1% TCCA bleaching at room temperature for 15 min.

### Comparison of bleaching ability of three oxidants

**Figure 1:**
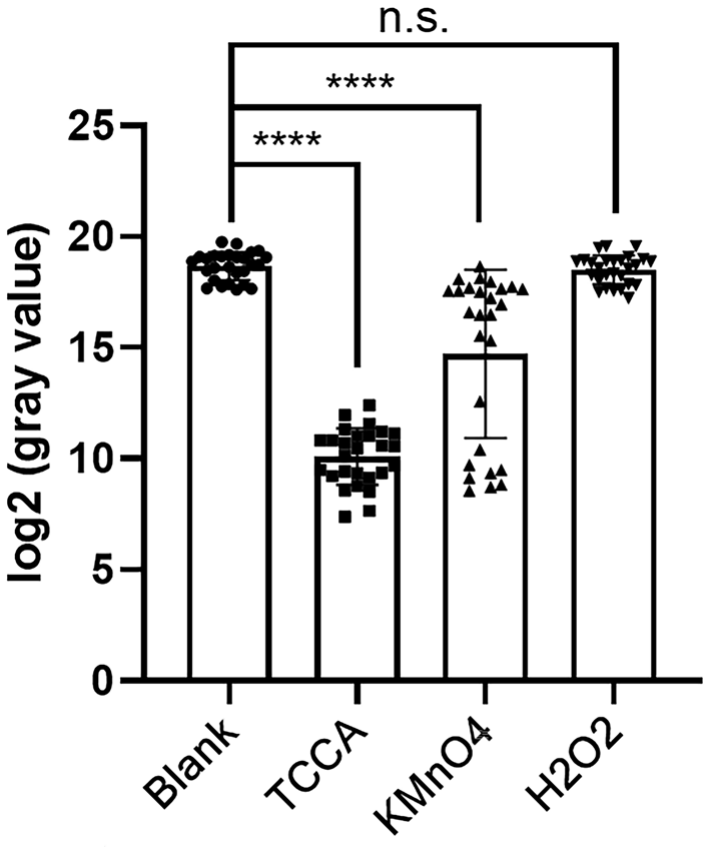
Gray values of 27 cases of tissues with high melanin content bleached by different oxidants for 15 min. The ordinate is the gray value converted by log, and the horizontal coordinate is the name of oxidant. Blank: undiscolored group; *TCCA* trichloroisocyanuric acid group, ****p < 0.0001, compared with blank; KMnO4: potassium permanganate group, ****p < 0.0001, compared with blank; *H*_*2*_*O*_*2*_ hydrogen peroxide group, p > 0.05, compared with blank. The data are presented as the mean ± SD.

The pathological sections of 27 tissues with a high melanin content (melanoma, *N* = 23; melanocytoma, *N* = 4) were treated with 0.5% potassium permanganate, 10% hydrogen peroxide, and 1% TCCA at room temperature for 15 min. The untreated blank control group was set up (gray value: 4.57 × 10^5^ ± 1.89 × 10^5^). The potassium permanganate group comprised 18 cases of melanin residue, in which the color became lighter (gray value: 1.23 × 10^5^ ± 1.16 × 10^5^). In the hydrogen peroxide group, all the tissues with high melanin content remained with heavy color (gray value: 4.06 × 10^5^ ± 1.78 × 10^5^). No obvious color difference was observed between the control and blank groups. All the 27 pathological sections in 1% TCCA group were purified (gray value: 1.52 × 10^3^ ± 1.24 × 10^3^). After analysis using GraphPad Prism 8 (Fig. 1), the color difference between the control, TCCA, and 0.5% potassium permanganate groups was statistically significant (*P* < 0.0001), and the hydrogen peroxide group showed no difference compared with the control group (*P* > 0.05)  (Supplementary figure 1).

### HE staining scheme after bleaching with 1% TCCA following normal tissue verification

**Figure 2:**
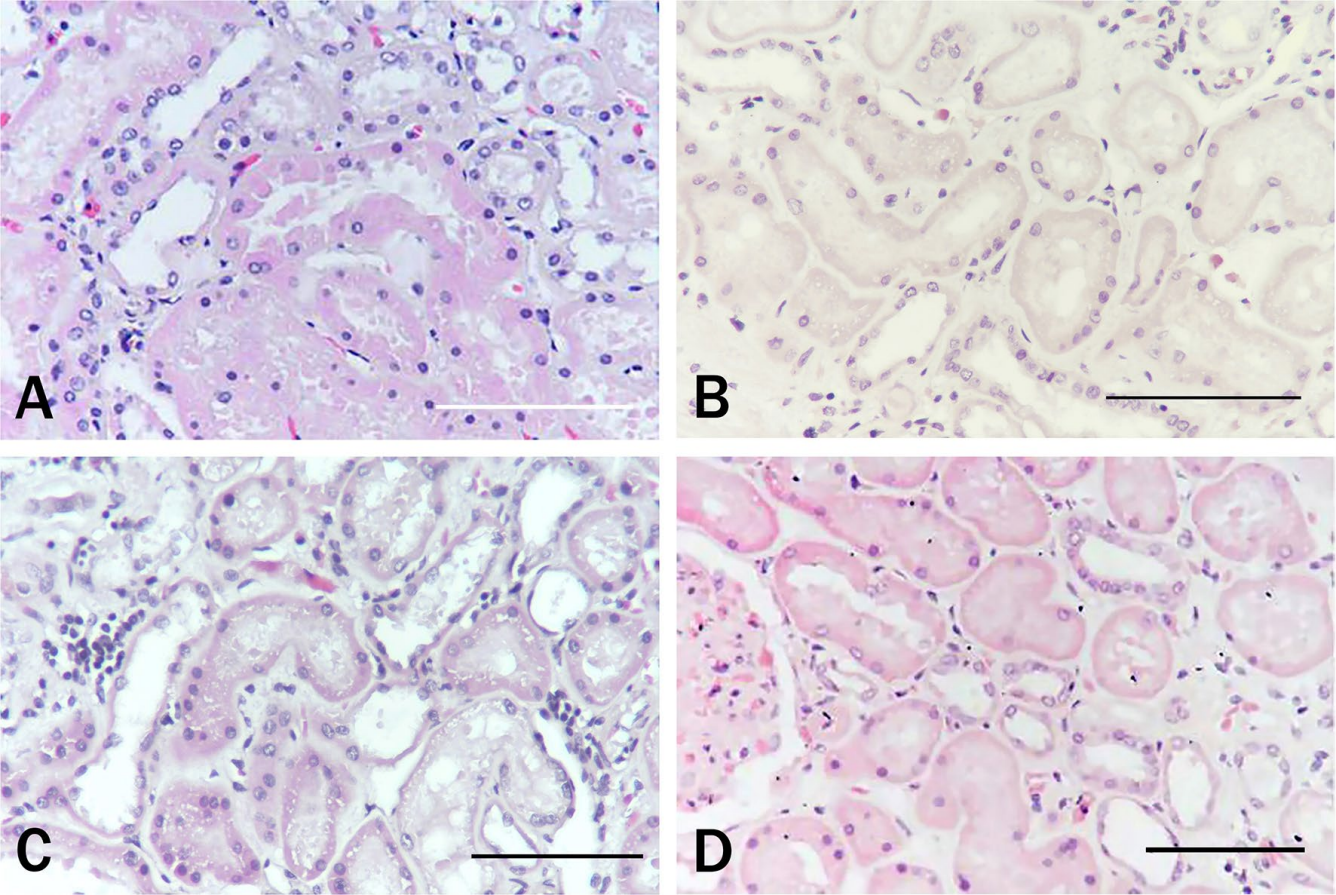
Comparison of normal control renal tissues treated with 1% TCCA for 15 min under different HE staining conditions: (**A**) untreated control, conventional HE staining; (**B–D**) HE staining after 1% TCCA treatment: (**B**) conventional HE staining; (**C**) extended time HE staining; (**D**) restored pH HE staining. Scale bar means 100 μm.

Nine normal tissues including kidney were set as the control group. Referring to HE standard score, the average score was 92.11 ± 1.27. The cytoplasm of the proximal tubules of the kidney tissue stained pink while the nucleus stained blue with a sharp contrast of red and blue (Fig. 2A). After bleaching with 1% TCCA, the HE staining scores in the experimental group were as follows.

#### HE scheme

In the experimental group, the kidney and other tissues were light stained with red and blue colors (Fig. 2B), A difference was found in the morphology score (23.33 ± 6.61 vs 92.11 ± 1.27, *P* < 0.05), but not up to the dyeing standard, in the experimental group compared with the control group.

#### Extended-time HE scheme

After knowing the HE stain rejection problem of oxidant TCCA, we simply extended the action time of the dye and expected the cells to have staining signals or signal enhancement, so as to realize morphological observation. For example, hematoxylin staining was extended to 10–30 min, and eosin staining was extended to 10–30 min. However, the result was still not ideal (cell nucleocytoplasmic comparison could not be achieved). After prolongation for 30 min, some renal tubules showed small cavities (Fig. 2C). The nuclear cytoplasmic contrast and morphology score of the "extended-time" group were statistically different (54.44 ± 7.26 vs 92.11 ± 1.27, *P* < 0.05) compared with those of the control group.

#### Restored pH HE scheme: nucleus

The cells were incubated with EDTA-Tris solution at pH 8–11 for 5, 10, and 20 min; the incubation time for pH 9.0 EDTA-Tris was 10 min. The hematoxylin staining showed dark blue small lymphocytes and blue macrophage nuclear chromatin; cytoplasm: the sections were incubated with 1%, 10%, and 30% glacial acetic acid for 1, 1.5, 2, and 2.5 min. The results showed no obvious color separation of hematoxylin within 1.5 min after incubating with 10% glacial acetic acid. The red layers of smooth muscle, collagen fibers, and red blood cells were seen under eosin staining for 5 min. A combination of two immersion solutions: The cells in the experimental group were incubated with pH 9.0 EDTA-Tris solution for 10 min, stained with hematoxylin for 10 min, incubated with 10% glacial acetic acid for 1.5 min, and stained with eosin for 5 min, The contrast between nucleus and cytoplasm was obvious. No statistically significant difference was found in the morphological score between the "Restored pH" group and the control group (88.89 ± 3.44 vs 92.11 ± 1.27, *P* > 0.05) (Fig. 2D).

### Application of clinical samples with different melanin contents

**Figure 3:**
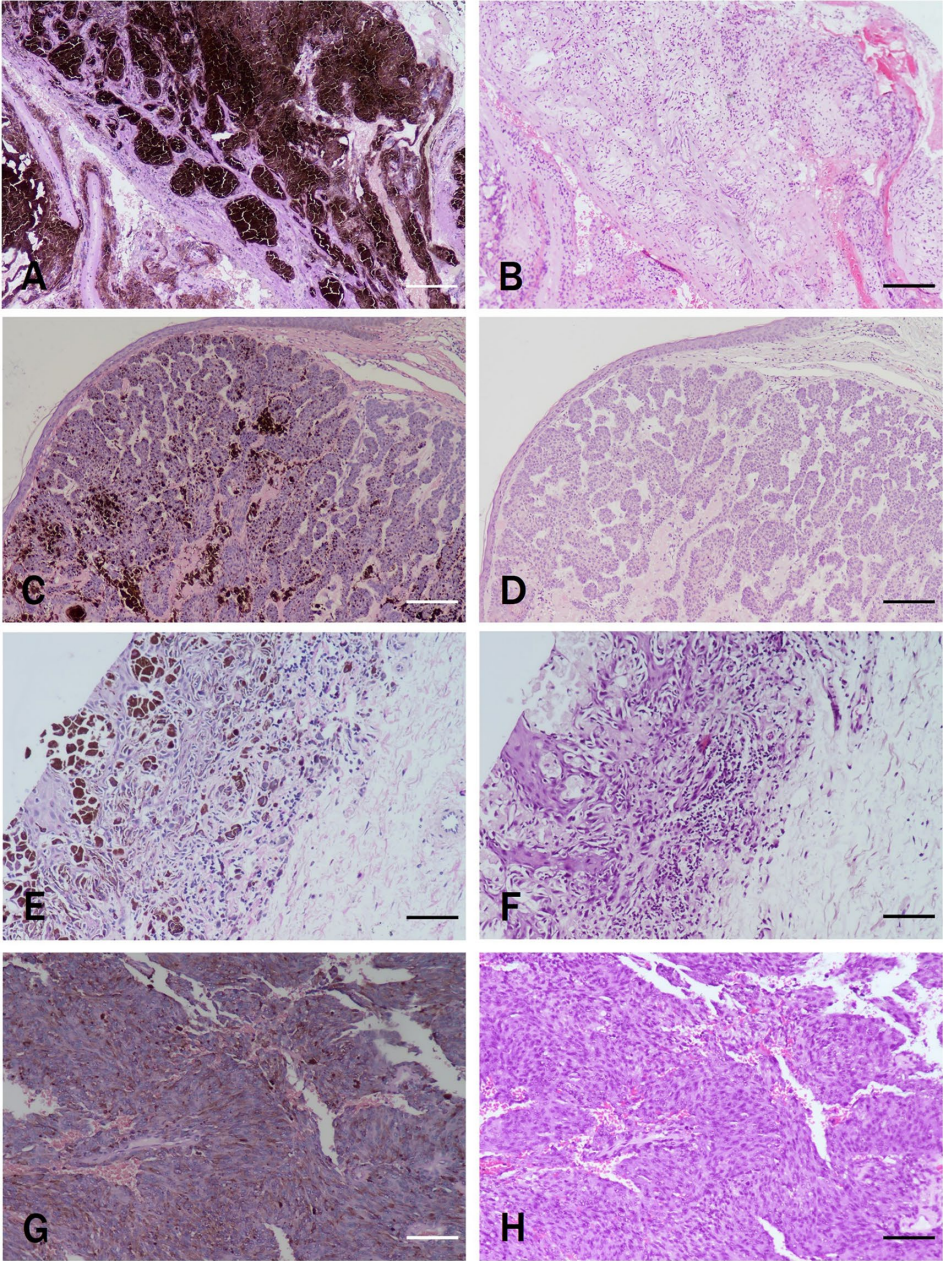
Different types of melanin after treatment with 1% TCCA typical pictures of pathological sections with different melanin types treated with 1% TCCA and stained with "Restored pH" HE method. (**A**) Central melanocytoma: conventional HE staining without melanin bleaching. (**B**) Central melanocytoma: HE staining of "Restored pH" after melanin bleaching. (**C**) Skin adnexal tumor: conventional HE staining without melanin bleaching. (**D**) Skin adnexal tumor: HE staining of "Restored pH" after melanin bleaching. (**E**) Compound nevus: conventional HE staining without melanin bleaching. (**F**) Compound nevus: HE staining of "Restored pH" after melanin bleaching. (**G**) Malignant melanoma: conventional HE staining without melanin bleaching. (**H**) Malignant melanoma: HE staining of "Restored pH" after melanin bleaching. Scale bar means 100 μm.

The pathological sections of 50 cases with melanin were incubated with 1% TCCA solution. The time of melanin bleaching in each tissue section was observed and recorded under a microscope. The bleaching time in the low-, medium-, and high-melanin content groups was less than or equal to the preset time of 15 min (Table 3). The melanin bleaching tissues were stained with “Restored pH HE scheme”. According to the HE staining scoring standard, the scores were all above 80 (Table 3). This study found that the staining after melanin removal could meet the needs of diagnosis (Fig. 3).

**Table 3:** Time required for complete melanin bleaching of 50 pathological sections with melanin in 1% TCCA solution.

Melanin content	Histologic type	Number	Complete melanin bleaching time (min)	Average staining score after melanin bleaching (point)
Low	Compound nevus, blue nevus, etc	N = 12	8.75 ± 0.75	87.17 ± 1.95
Medium	Blue nevus	N = 5	12.40 ± 1.14	84.4 ± 2.51
	Cutaneous melanoma	N = 6	12.17 ± 0.75	83.83 ± 4.49
High	Cutaneous melanoma	N = 23	14.62 ± 1.04	80.04 ± 2.5
	Meningeal melanocytoma	N = 4	14.00 ± 1.41	83.5 ± 2.65

### Comparison of morphological diagnostic scores before and after 1% TCCA bleaching

**Figure 4:**
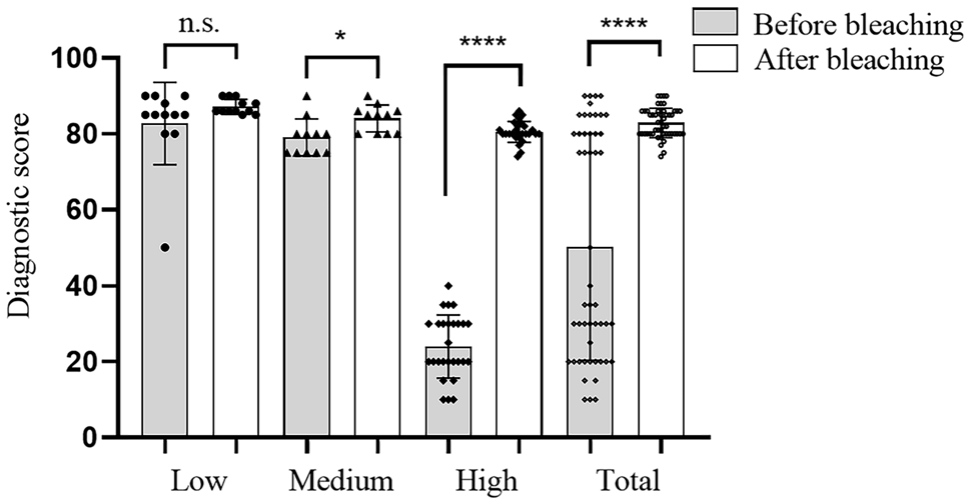
Comparison of diagnostic scores of 50 pathological sections with melanin before and after bleaching, Low, medium and high melanin groups (n = 12, 11, 27). In the low melanin content group (n = 12), there was no difference in the diagnostic value before and after bleaching (P > 0.05); In the medium melanin content group (n = 11), there was difference in the diagnostic value before and after bleaching (*P < 0.05); In the high melanin content group (n = 27), there was difference in the diagnostic value before and after bleaching (****P < 0.0001); The data are presented as the mean ± SD.

In pathological sections of 50 cases with melanin, 3 pathologists compared the mean value of morphological HE staining before and after bleaching with 1% TCCA in a double-blind manner. The score was from 0 to 100 points according to the final pathological diagnosis (gold standard). A significant difference was found in the overall diagnostic score before and after bleaching (Fig. 4; 50.26 ± 29.85 vs 82.92 ± 3.91, *P* < 0.0001). According to the melanin content, no difference in the diagnostic score before and after bleaching in the low–melanin content group (*n* = 12) (82.75 ± 10.86 vs 87.17 ± 1.95, *P* > 0.05). However, a significant differences was found between the samples with a medium (*n* = 11) and high (*n* = 27) melanin content before and after bleaching (79.09 ± 4.91 vs 84.09 ± 3.56, *P* < 0.05, 24.07 ± 8.32 vs 80.56 ± 2.76, *P* < 0.0001). The results showed that in the middle– and high–melanin content groups, the diagnosis score could be better improved by removing melanin with 1% TCCA (Supplementary figure 2).

### Effect of melanin removal with TCCA on immunohistochemistry

Previous experimental data showed that the tissues with high melanin content were bleached after immunohistochemical staining with different concentrations of TCCA. It was found that 8% TCCA was the best reaction concentration^8^. By bleaching the control tissue sections without melanin for 20 min, all kinds of antibodies (Class 3 and Class 1) had accurate positioning and consistent intensity, referring to the key dyeing threshold or lower dyeing limit components (Table 4). When the immersion time was prolonged to 20 h, the lymphofollicular nuclear signal of ER disappeared in the tonsil^9^. After 24 h, the signal of ovarian PR weakened. After 36 h, the signal of HER-2-positive staining was attenuated by grade 1^10^. The test results of the tolerance time of other antibodies are shown in Table 4.

**Table 4:** Immunohistochemical marker tolerance to 8% TCCA bleaching duration.

Markers	Dyeing threshold	Tolerance time (h)
ER	Tonsil follicular lymphocytes	20
PR	Ovary	24
HER-2	2 + Breast cancer	36
CD20	Liver plasma cell	72
CD117	Appendix Cajal cells	72
ALK	Appendix ganglion cells	72
PD-L1	Amygdala macrophages	48
Ki67	Tonsil follicles	48
S100	Amygdala macrophages	77
SOX10	Appendix smooth muscle cells	78
CK	Liver, kidney tubules	78
β-catenin	Liver	72
MLH1	Appendix smooth muscle cells	72

**Figure 5:**
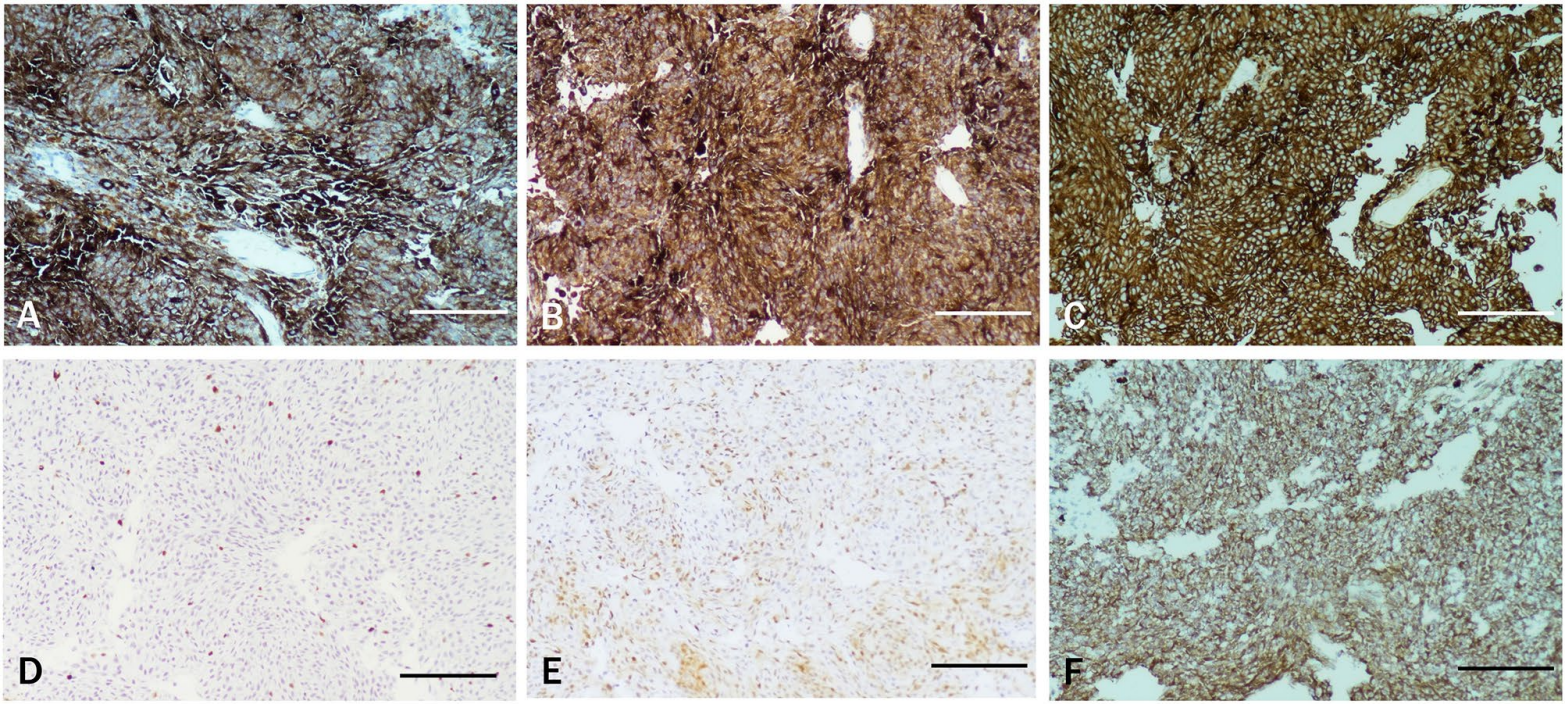
Immunohistochemical staining was performed on meningeal melanoma tissue. (**A,D**) were stained with ki67; (**B,E**) were stained with S100; (**C,F**) were stained with β-catenin. (**D–F**) were treated with 8% TCCA. Scale bar means 100 μm.

Immunohistochemical staining was performed on meningeal melanoma tissue. For this, 8% TCCA solution was used to remove melanin after Ki67 (nuclear positive, Fig. 5A,D), S100 (nucleocytoplasmic positive, Fig. 5B,E), and β-catenin (membrane positive, Fig. 5C,F) immunohistochemical staining. In the melanin unbleached group, a large amount of melanin was covered. After bleaching with TCCA, the staining and positioning of each marker were accurate and the background was clear. The results showed that after immunohistochemistry, the melanin-containing tissues were treated with 8% TCCA to remove melanin. It could not only effectively remove melanin but also did not affect DAB products.

### *Effect of melanin bleach with TCCA on EBER *in situ* hybridization*

**Figure 6:**
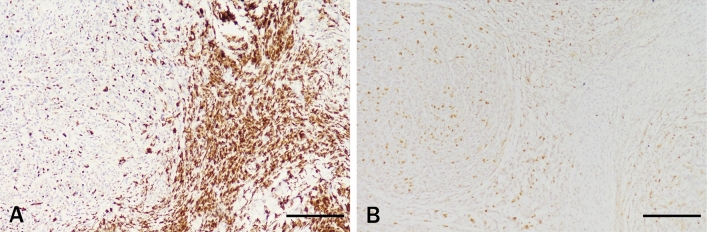
EBER positive tissue. (**A**) EBER-ISH staining without melanin bleaching. (**B**) was treated with 8% TCCA. Scale bar means 100 μm.

In this study, three cases of EBER-positive tumors containing melanin were bleached with 8% TCCA for 20 min. The results showed that the nuclear localization of the EBER signal in tumor cells was clear, and the background was clean (Fig. 6). The results showed that 8% TCCA did not affect EBER in situ hybridization.

## Discussion

TCCA has stable chlorine activity at room temperature (about 2 h) as reported^11,12^. In this study, three cases of tissues with a high melanin content (more than 50% of tumor nuclei or plasma) were bleached at room temperature. A total of 27 cases of high melanin content were bleached with different oxidants for 15 min: the depigmentation of 0.5% potassium permanganate was not complete, and it took a long time for some cases to depigment. The depigmentation time of 0.25% potassium permanganate was about 30 min, which was consistent with a literature report^13^. Further, 10% hydrogen peroxide barely changed in 15 min unless after 12–48 h at room temperature^14^. However, it was not clear that higher concentrations could get better HE-stained sections^1,5^. Since HE staining was performed with general microscopic slides, the higher the concentration of TCCA, the easier it was to cause the paraffin tissue to drop off. Also, 1% TCCA was bleached completely in 15 min at room temperature after repeated experiments. Therefore, 1% TCCA was set as the working concentration.

The morphological changes in melanin cases before and after bleaching could not be observed. Hence, the establishment of normal tissues such as kidneys and pancreas as standard controls for HE staining might be conducive to the comparison of histomorphology before and after bleaching^11^. After bleaching with 1% TCCA, normal control tissues such as kidneys were difficult to be colored using a conventional HE scheme; the color was light and lacked contrast. Using the “extended-time” HE scheme, the staining was gradually deepened with the increase in HE time, but the contrast was partial or purplish. The contrast balance could not be achieved even after the extension of the total time to greater than 1 h; therefore, it was considered that the oxidizer might cause tissue rejection. In the "Restored pH" scheme, EDTA-Tris alkaline retrieval solution with pH 8.0–11.0 was used for incubation: EDTA-Tris with pH 9.0 was incubated at room temperature for 10 min and hematoxylin staining for 10 min, and the nuclear components were observed. The small lymphocytes were stained dark blue, and the macrophages showed fine light blue chromatin, suggesting that this condition met the needs of nuclear staining polarity. In the experimental test of EDTA-Tris solution compensation, the pH should be the same as that of the cell retrieval liquid phase (pH 9.0); otherwise, it is easy to drop from the slide and affect eosin staining. After incubating with different concentrations of glacial acetic acid solution at room temperature, it was found that the smooth muscle was stained dark red, the collagen fibers were stained pink, and the red blood cells were stained cherry red after incubating with 10% glacial acetic acid solution for 1.5 min and eosin for 2–5 min. The proximal tubules were stained pink, suggesting that 10% glacial acetic acid was beneficial to compensate for the polarity of cytoplasmic components. It was also found that 30% glacial acetic acid could cause obvious differentiation and discoloration of hematoxylin, while 10% glacial acetic acid was relatively mild and the discoloration was slow. The "Restored pH" program combined with pH compensation of two chemical solutions had an obvious effect on the staining of tissue sections after bleaching. According to the judgment of more than one attending doctor, the morphology of 1% TCCA before and after bleaching had no change, which met the needs of diagnosis. Therefore, we chose "restore pH" HE scheme as the staining method after TCCA melanin removal.

The proven morphological staining scheme of 1% TCCA was applied to 50 pathological sections with melanin. The pathologists could see clear tumor cell morphology (round, spindle shape), distribution, cell polymorphism degree, cytoplasm appearance (large or rare), nuclear characteristics (size, shape, atypical), nucleolar evidence, mitosis quantity, and inflammatory response of each field of vision. Also, the staining met the requirements of the diagnosis of pathological tissue morphology. It was consistent with the conclusion of similar research^15^. In some cases with a high melanin content, the doctors give different diagnoses and differential diagnostic ideas (for example, one case of melanoma is shown in Fig. 3A. The possibility of malignant melanoma was considered when no bleaching occurred, and central melanocytoma/nonmelanoma was considered after bleaching). It was suggested that melanin bleaching technique had a greater effect on the samples of medium and high melanin types. After bleaching, immunohistochemical and molecular detection were still needed to make a definite diagnosis, which can avoid misdiagnosis and medical disputes^18^. This also prompted us to further research the effect of TCCA on immunohistochemistry and molecular experiments. In immunohistochemical experiments, Ki67 staining and bleaching were performed on 5 cases of high melanin tissues: the concentration of TCCA solution was inversely proportional to the reaction time (After Ki67 staining, 5 cases of high melanin tissues were bleached: the concentration of TCCA solution was inversely proportional to the reaction time (1%, 2%, 4%, 8%,16% TCCA bleaching time was 72.6 ± 5.3 min, 46.9 ± 4.4 min, 31.5 ± 3.9 min, 13.6 ± 1.4 min and 4.1 ± 1.1 min respectively). This part of the results has been reported in our previous study^8^. Considering that 16% TCCA bleaching has short reaction time but unstable concentration, 8% TCCA solution is selected as the optimal reaction concentration.

Molecular detection is indispensable for the diagnosis of melanin-rich tumors^16^. Whether the new bleaching scheme is suitable for molecular detection is worth exploring. The EBER signal was not affected by 8% TCCA bleaching. EBER is also based on the DAB in situ color rendering method. In this study, it was found that TCCA would lead to DNA fragmentation and loss of HER2 fish signal, etc. because the oxidant would damage the nucleic acid, negative results or bad results were obtained, so the text did not include. Nucleic acids are sensitive to acids^17^, and TCCA is essentially an acid, which inevitably leads to DNA damage. In FISH detection, melanin only causes poor DAPI staining and does not interfere with fluorescent staining signal.

In conclusion, the modified scheme of tissue bleaching and HE staining for TCCA established in this study was based on the timeliness and quality control of the clinical operation and realized the effective pathological diagnosis of melanin-covered disease. 1% TCCA was especially suitable for HE staining in the diagnosis of benign and malignant melanoma with medium and high melanin coverage. 8% TCCA was suitable for methodologies that rely on DAB coloration, such as IHC and EBER. The solution preparation method was simple, economical, safe, and easy to operate. After verification of normal tissues and confirmation of clinical samples, this study established a scheme for treating pathological sections with different dilutions of TCCA. It provided a repeatable technical method for the diagnosis and differential diagnosis of medium- and high-melanin disease, and provided the possibility for follow-up research on immunohistochemical differential diagnosis (Supplementary information).

## Supplementary Information

Supplementary Figure 1.

Supplementary Figure 2.

## Data Availability

All data generated during this study are included in this published article and its supplementary information files.
