# Supplementary material for: Construction of hematoxylin–eosin, immunohistochemistry, and EBER-ISH methodology after trichloroisocyanuric acid treatment in melanin-containing tissues
Source: Sci Rep. 2022 Sep 26;12:16062. doi: 10.1038/s41598-022-20535-7 (PMC9512931; doi:10.1038/s41598-022-20535-7)

Supplementary figure 1.

Gray values converted by log of 27 cases of tissues with high melanin content bleached by different oxidants for 15mins.

|  | Blank | TCCA | KMnO4 | H2O2 |
| --- | --- | --- | --- | --- |
| 1 | 18.461 | 9.134 | 9.719 | 18.038 |
| 2 | 19.092 | 10.564 | 17.971 | 18.584 |
| 3 | 17.83 | 9.364 | 8.547 | 17.571 |
| 4 | 18.447 | 7.384 | 8.833 | 18.282 |
| 5 | 17.861 | 8.581 | 9.142 | 17.817 |
| 6 | 18.366 | 10.134 | 16.948 | 18.236 |
| 7 | 19.077 | 7.658 | 8.718 | 18.823 |
| 8 | 19.007 | 11.973 | 16.587 | 18.884 |
| 9 | 19.109 | 10.827 | 18.662 | 18.925 |
| 10 | 18.766 | 12.413 | 17.706 | 18.55 |
| 11 | 19.682 | 9.351 | 9.494 | 19.483 |
| 12 | 19.184 | 9.418 | 17.549 | 18.989 |
| 13 | 17.756 | 9.222 | 16.491 | 17.507 |
| 14 | 19.77 | 10.475 | 17.552 | 19.578 |
| 15 | 19.357 | 11.578 | 17.735 | 19.102 |
| 16 | 18.739 | 10.694 | 10.4 | 18.904 |
| 17 | 19.305 | 9.674 | 17.51 | 19.574 |
| 18 | 18.642 | 11.015 | 16.479 | 18.718 |
| 19 | 17.619 | 11.032 | 17.242 | 17.611 |
| 20 | 18.594 | 8.785 | 17.649 | 17.238 |
| 21 | 19.08 | 11.326 | 12.563 | 18.89 |
| 22 | 17.673 | 10.819 | 18.135 | 17.681 |
| 23 | 19.041 | 11.226 | 17.642 | 18.9 |
| 24 | 17.657 | 8.52 | 18.099 | 17.865 |
| 25 | 18.03 | 10.6 | 9.347 | 18.283 |
| 26 | 18.878 | 11.143 | 15.315 | 18.335 |
| 27 | 19.145 | 9.482 | 15.532 | 18.954 |

Original picture on next page, see materials and methods for details.

Case 1

Case 4

Case 7

Case 6

Case 5

Case 9

Case 11

Case 14

Case 21

Case 26


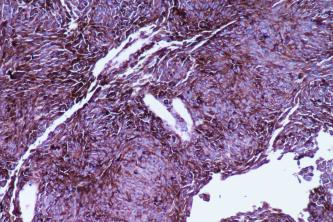

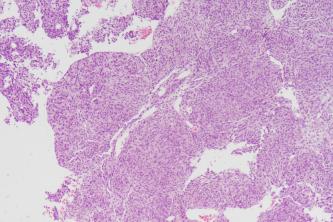

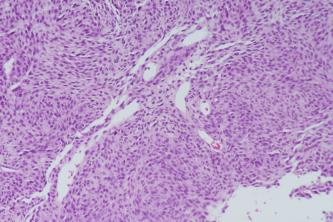

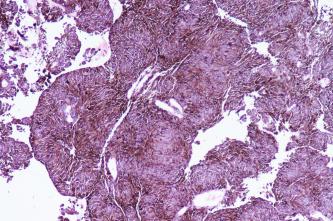

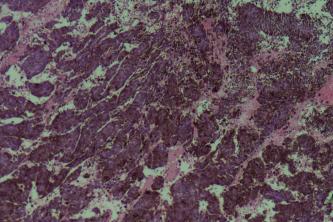

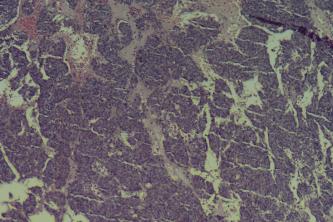

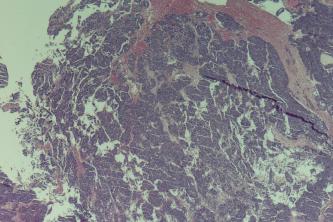

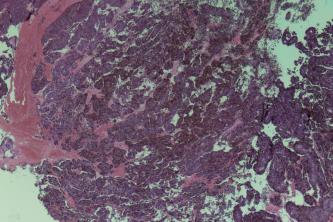


control

KMnO_4_

H_2_O_2_

TCCA


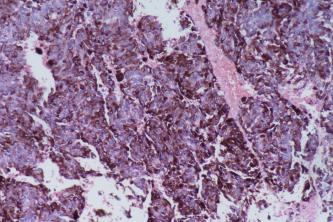

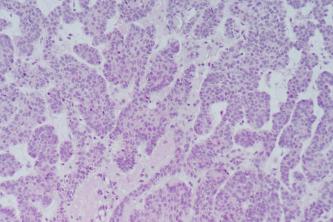

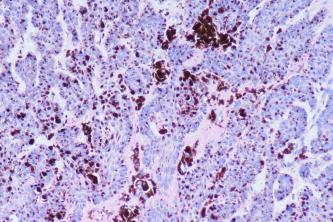

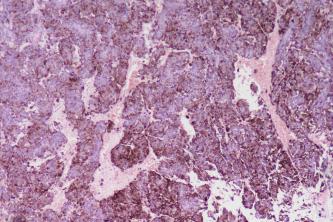

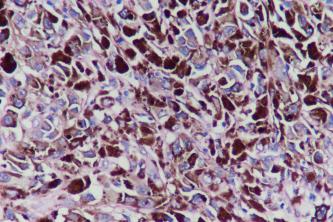

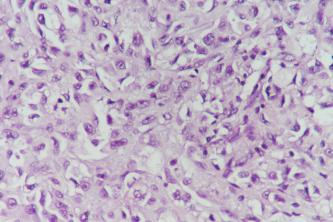

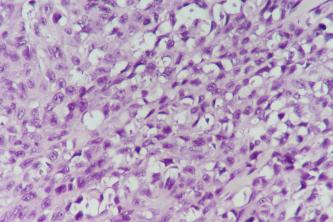

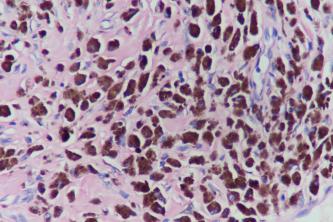

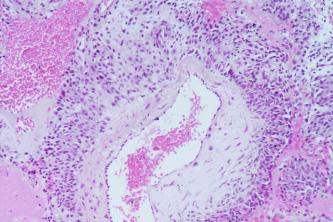

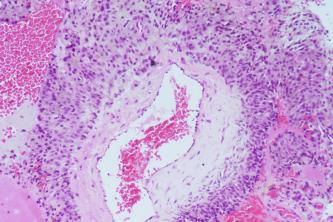

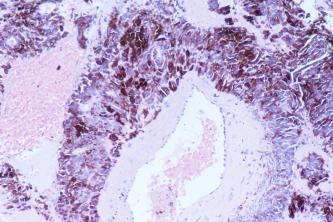

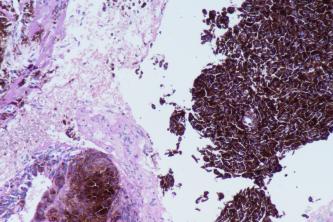

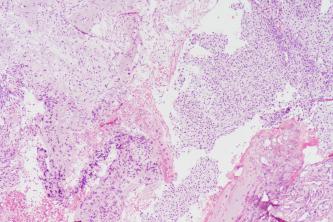

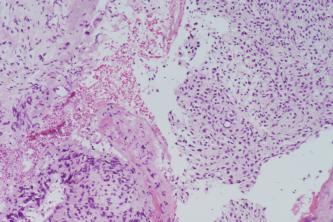

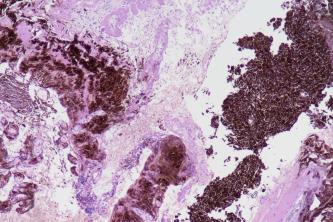

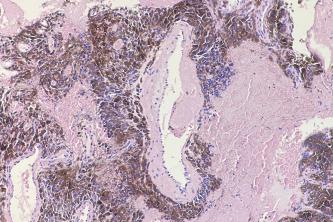

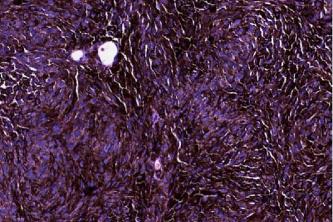

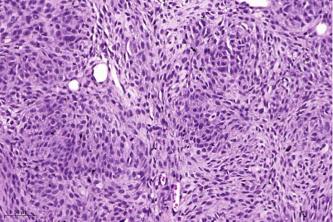

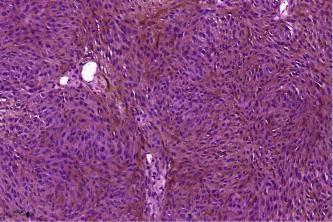

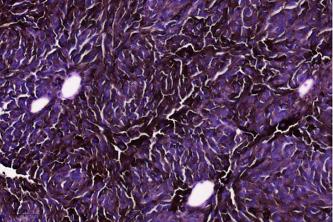

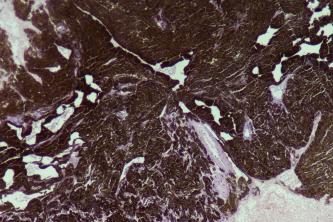

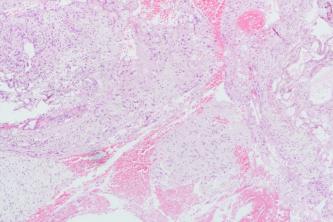

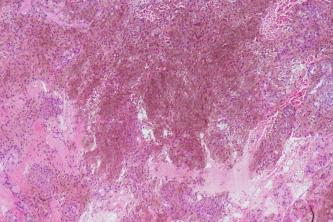

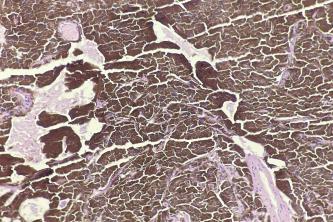

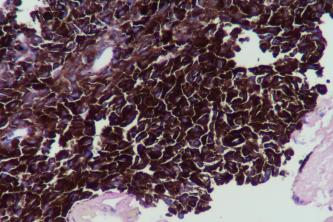

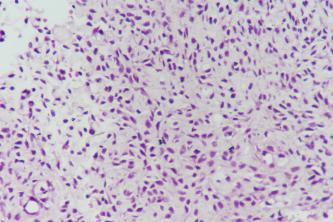

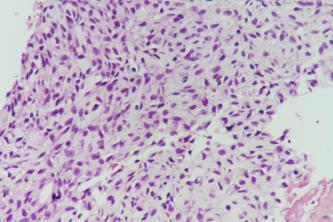

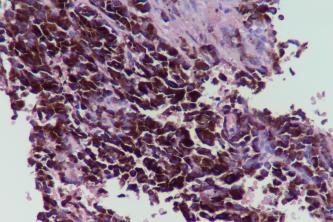

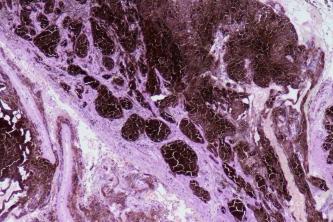

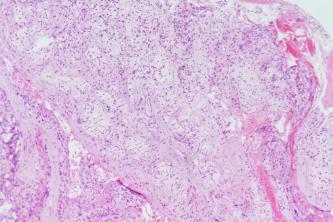

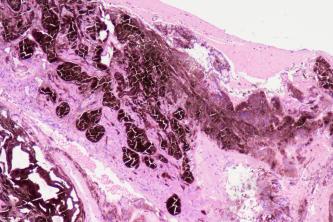

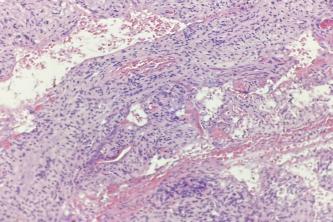

Supplement: Supplementary file 1 — Supplementary Figure 1. [file 41598_2022_20535_MOESM1_ESM.docx]
