# Supplementary material for: Construction of hematoxylin–eosin, immunohistochemistry, and EBER-ISH methodology after trichloroisocyanuric acid treatment in melanin-containing tissues
Source: Sci Rep. 2022 Sep 26;12:16062. doi: 10.1038/s41598-022-20535-7 (PMC9512931; doi:10.1038/s41598-022-20535-7)

Supplementary figure 2.

Comparison of diagnostic scores of 50 cases containing melanin before and after bleaching, Low, medium and high melanin groups

| Num | | Low before | Low after | Num | Medium before | Medium after | Num | High before | High after |
| --- | --- | --- | --- | --- | --- | --- | --- | --- | --- |
| 1 | 80 | | 85 | 1 | 80 | 86 | 1 | 10 | 80 |
| 2 | 85 | | 86 | 2 | 80 | 85 | 2 | 20 | 80 |
| 3 | 88 | | 88 | 3 | 80 | 85 | 3 | 20 | 81 |
| 4 | 90 | | 90 | 4 | 75 | 80 | 4 | 30 | 80 |
| 5 | 85 | | 86 | 5 | 85 | 86 | 5 | 10 | 78 |
| 6 | 80 | | 86 | 6 | 80 | 88 | 6 | 20 | 85 |
| 7 | 50 | | 88 | 7 | 75 | 85 | 7 | 20 | 80 |
| 8 | 90 | | 90 | 8 | 90 | 90 | 8 | 30 | 81 |
| 9 | 85 | | 86 | 9 | 75 | 80 | 9 | 40 | 83 |
| 10 | 85 | | 86 | 10 | 75 | 80 | 10 | 30 | 75 |
| 11 | 85 | | 85 | 11 | 75 | 80 | 11 | 10 | 80 |
| 12 | 90 | | 90 |  |  |  | 12 | 20 | 80 |
|  |  | |  |  |  |  | 13 | 30 | 81 |
|  |  | |  |  |  |  | 14 | 20 | 82 |
|  |  | |  |  |  |  | 15 | 35 | 79 |
|  |  | |  |  |  |  | 16 | 20 | 79 |
|  |  | |  |  |  |  | 17 | 30 | 80 |
|  |  | |  |  |  |  | 18 | 15 | 80 |
|  |  | |  |  |  |  | 19 | 15 | 82 |
|  |  | |  |  |  |  | 20 | 20 | 77 |
|  |  | |  |  |  |  | 21 | 30 | 84 |
|  |  | |  |  |  |  | 22 | 35 | 74 |
|  |  | |  |  |  |  | 23 | 25 | 80 |
|  |  | |  |  |  |  | 24 | 20 | 80 |
|  |  | |  |  |  |  | 25 | 30 | 83 |
|  |  | |  |  |  |  | 26 | 30 | 86 |
|  |  | |  |  |  |  | 27 | 35 | 85 |

Original picture on next page, see materials and methods for details.


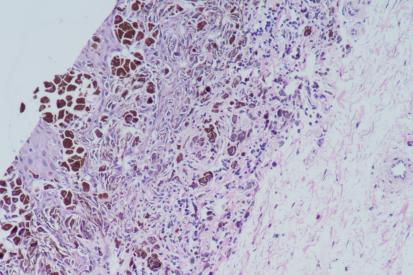

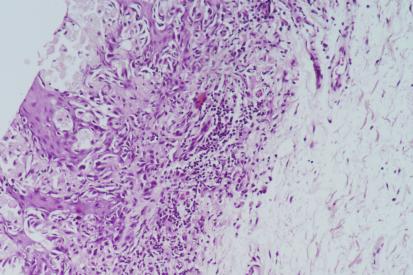

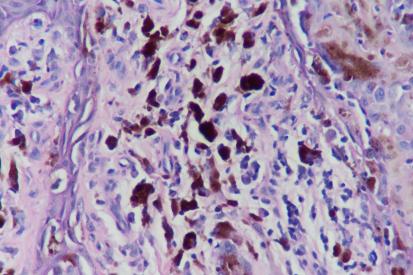

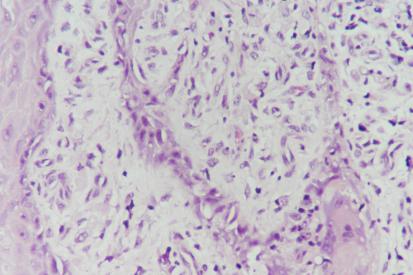


low before

low after

case 1

case 8

case 7


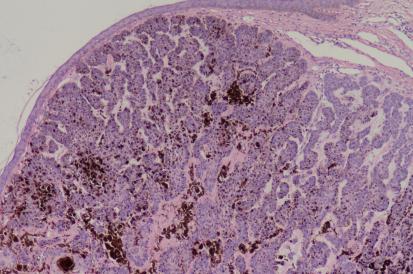

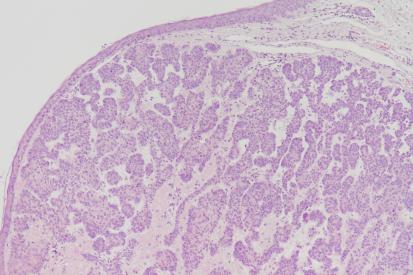


case 1

case 3

case 2

medium before

medium after


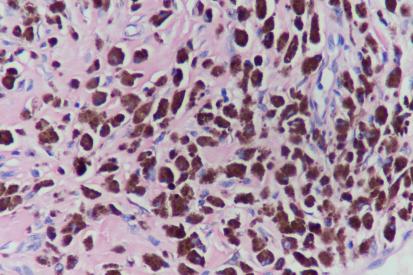

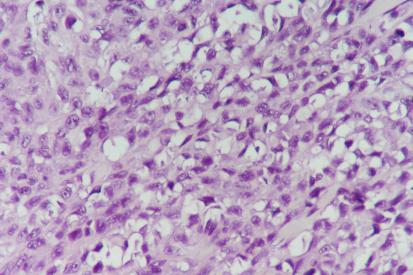

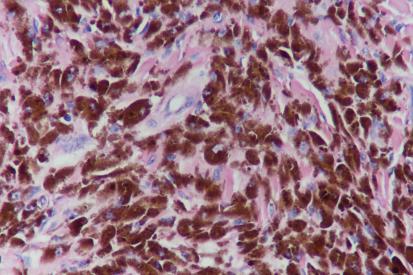

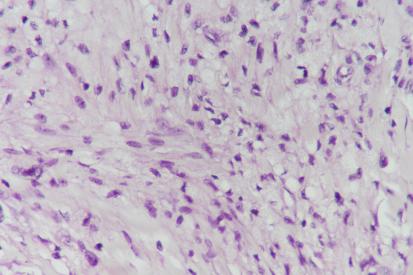

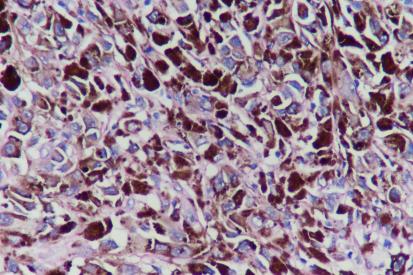

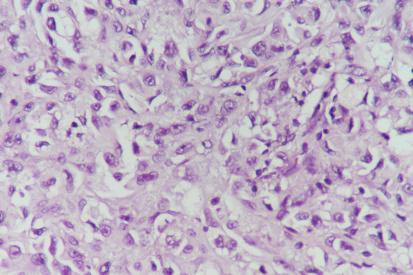

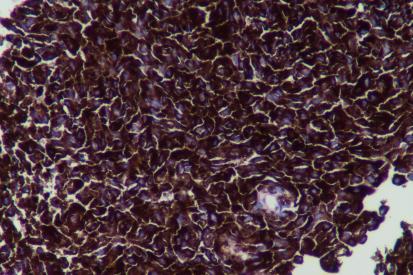

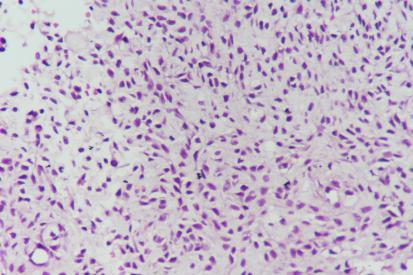

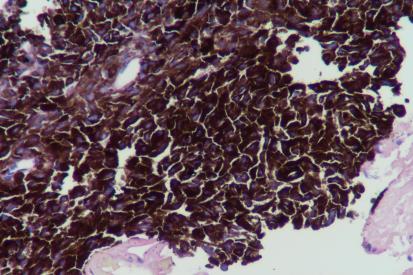

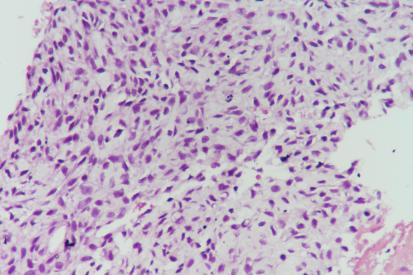

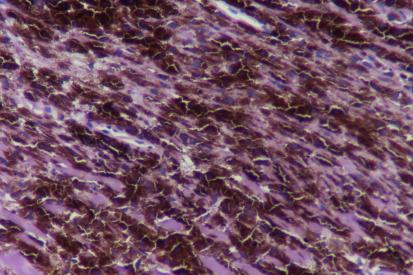

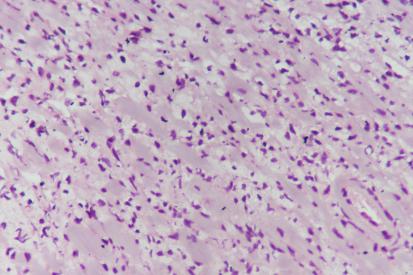

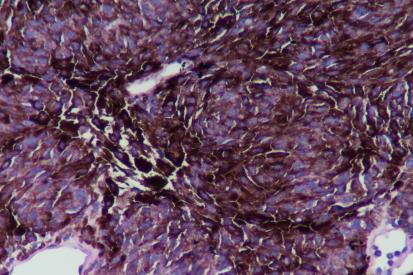

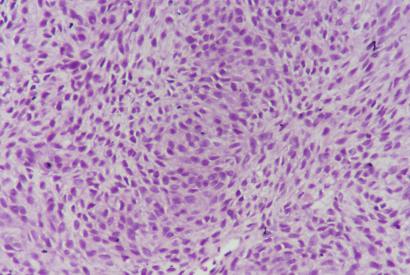


high

before

high

after

case 2

case 4

case 3

case 1

high

before

high

after

case 25

case 27

case 26

case 23


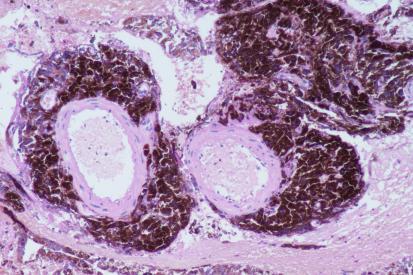

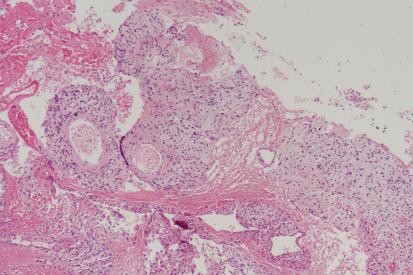

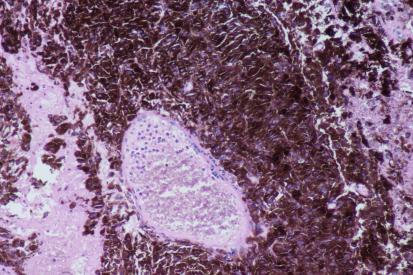

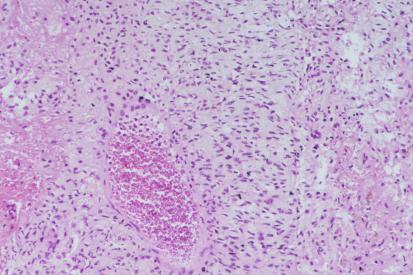

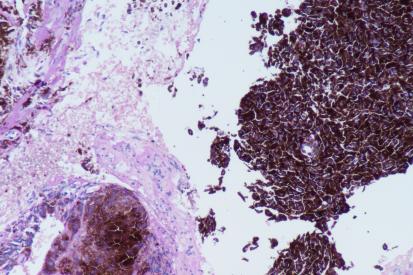

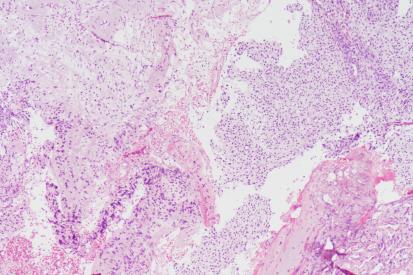

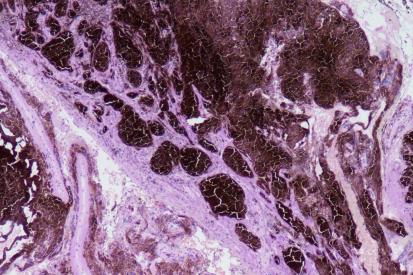

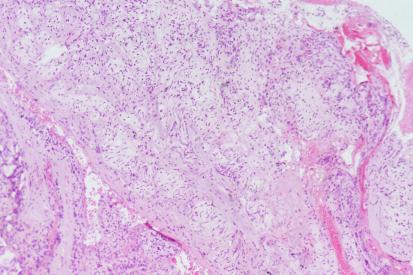


high

before

high

after

case 17

case 22

case 18

case 15


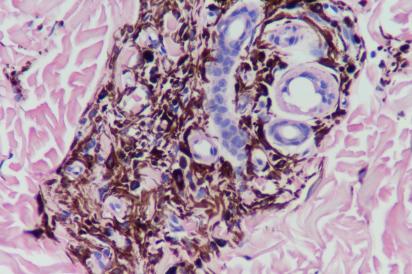

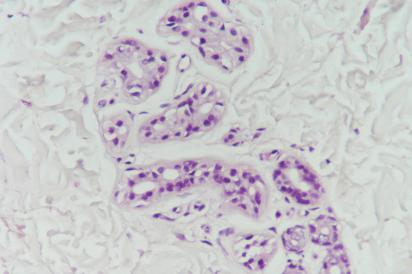

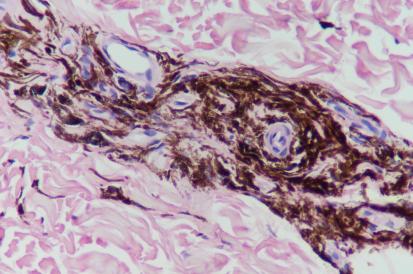

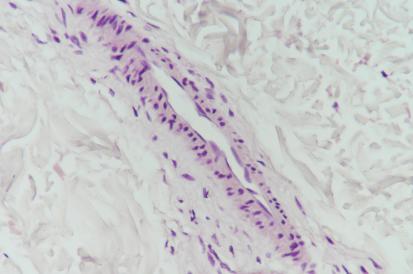

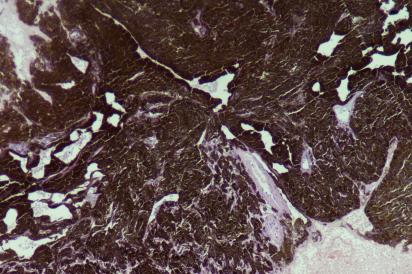

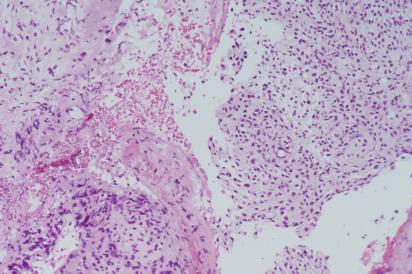

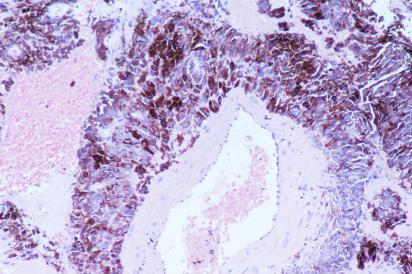

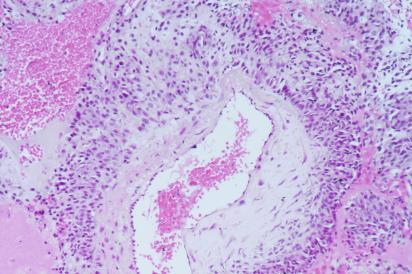


high

before

high

after

case 10

case 13

case 11

case 9


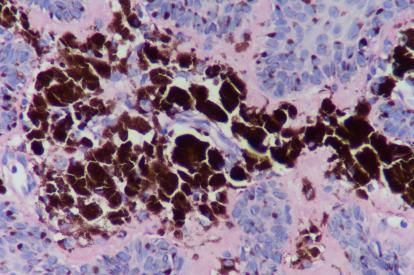

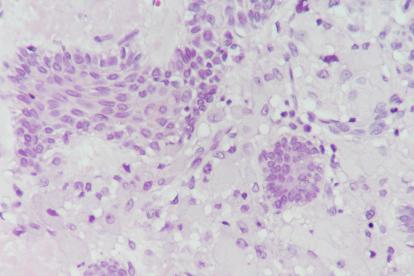

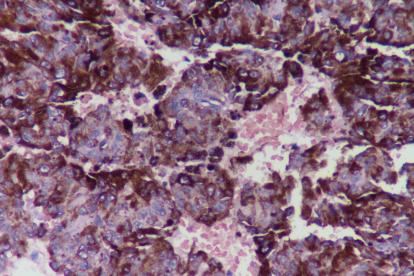

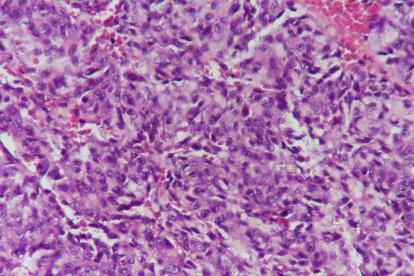

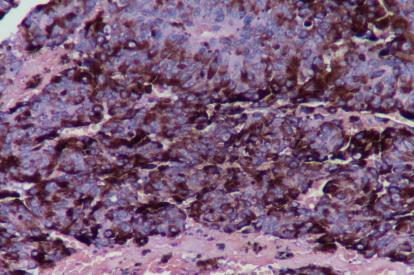

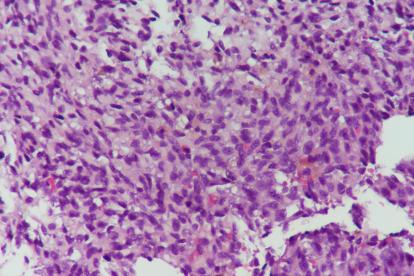

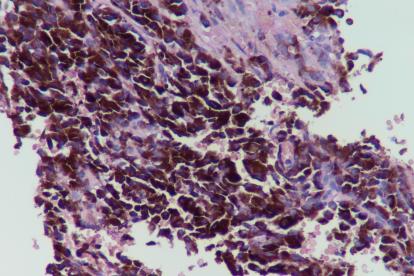

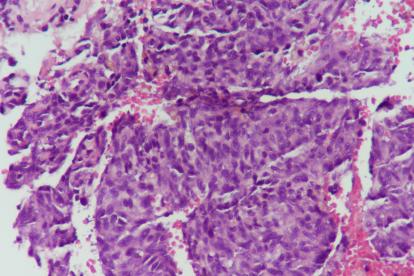

Supplement: Supplementary file 2 — Supplementary Figure 2. [file 41598_2022_20535_MOESM2_ESM.docx]
